# Detection of specific IgE against linear epitopes from Gal d 1 has additional value in diagnosing hen’s egg allergy in adults

**DOI:** 10.1111/cea.13730

**Published:** 2020-09-24

**Authors:** Anna M. Ehlers, Henny G. Otten, Eva Wierzba, Ulrike Flügge, Thuy‐My Le, André C. Knulst, Waltraud Suer

**Affiliations:** ^1^ Center for Translational Immunology University Medical Center Utrecht, Utrecht University Utrecht Netherlands; ^2^ Department of Dermatology/Allergology University Medical Center Utrecht, Utrecht University Utrecht Netherlands; ^3^ Euroimmun AG Lübeck Germany

**Keywords:** food allergy, hen, IgE, linear epitopes's egg

## Abstract

**Background:**

Although hen's egg allergy is more prevalent in children, up to 0.6% of adults from different European countries suffer from a persistent or newly onset hen's egg allergy, making accurate diagnosis in adults necessary. However, sensitization to hen's egg extracts, components and linear epitopes is solely studied in children.

**Methods:**

Hen's egg allergic (n = 16) and tolerant (n = 19) adults were selected by sensitization towards recombinant components rGal d 1 and/or 3. Sensitization profiles towards egg white and yolk extract and the native components Gal d 1, 2, 3 and 4 were respectively evaluated with the ImmunoCAP or the EUROLINE system. Characterization of linear epitopes was performed with a peptide microarray containing 15mer peptides representing the entire sequence of mature Gal d 1 and 3.

**Results:**

Overall, sIgE titres against hen's egg extracts and single components overlapped largely between allergic and tolerant adults. Although the median sIgE/sIgG4 ratio to Gal d 1 was increased in allergic adults, the range was comparable between both groups. Clinically relevant sensitization to Gal d 1 was confirmed by sIgE‐binding to the linear epitopes aa30‐41, aa39‐50 or aa84‐95 in 6/13 allergic adults, mainly suffering from objective symptoms. In comparison, these epitopes were recognized by 1/15 tolerant patient. Only a few linear epitopes were detected for Gal d 3, suggesting a greater importance of conformational epitopes for the recognition of Gal d 3.

**Conclusion and Clinical Relevance:**

Specific IgE‐binding to linear epitopes of Gal d 1 is highly specific in identifying hen's egg allergic adults with objective symptoms.

## 1 INTRODUCTION

Hen's egg is known as a major cause of food allergic reactions in children, and this allergy is often outgrown by the age of five. Nevertheless, the prevalence of hen's egg allergy in adults average 0.02 to 0.6% across European countries.^1^, ^2^ Although hen's egg allergy in adulthood is predominantly a persistent allergy developed in childhood, it can also be newly developed later in life.^3^ In a study conducted in the United States, 29% of all hen's egg allergic adults suffered from an adult‐onset allergy.^4^

Diagnosis of hen's egg allergy is comprised of anamnesis, skin prick test, measuring sIgE and food challenges. Food challenges are the gold standard, but they are burdensome, expensive and require dedicated hospital facilities and personnel. To avoid or replace food challenges, intensive research has been performed to improve the diagnostic value of sIgE measurements in hen's egg allergic children. In a systematic review, an evaluation of sIgE measurements towards egg white extract in children ranging from infants to adolescents showed an overall sensitivity of 0.93, but only a specificity of 0.49.^5^

Component‐resolved diagnostics improved the accuracy of sIgE measurement in several food allergies.^6^ Major allergenic components of hen's egg white, which is responsible for most of its allergenicity, are ovomucoid (Gal d 1), a thermo‐stable allergen, ovalbumin (Gal d 2), ovotransferrin (Gal d 3) and lysozyme (Gal d 4). Ovomucoid, the major allergen of egg white, is by far the most studied component in relation to hen's egg allergy in children and although ovomucoid is classified as a prognostic marker for persistent hen's egg allergy, its superior role compared to egg white extract has been debated.^7^

Patients’ sera contain polyclonal IgE antibodies recognizing a broad range of epitopes comprised of either sequential residues of the amino acid sequence (linear) or amino acids closely located upon folding (conformational). Epitope mapping approaches aim to identify clinically relevant epitopes which are undetectable by measuring sIgE against extracts or full‐length single components. In hen's egg allergy, linear epitope mapping of Gal d 1 identified epitopes (aa 1‐10, aa 11‐20, aa 47‐56 and aa 113‐122) exclusively recognized by children with persistent hen's egg allergy.^8^ Comparable allergenic parts (aa 1‐10, aa 11‐20 and aa 47‐56) were described as immunodominant linear epitopes by several other studies.^9^, ^10^, ^11^

So far, the impact of sIgE titres to hen's egg components and sIgE‐binding to their linear epitopes on discriminating between clinically relevant and irrelevant sensitization is poorly studied in hen's egg allergic and tolerant adults. To this end, we evaluated sensitization patterns and sIgE titres to hen's egg components (Gal d 1, 2, 3 and 4) in allergic and tolerant, but sensitized adults. Since Gal d 1 is known as the most important single component for diagnosing hen's egg allergy in children, recognition of linear epitopes derived from Gal d 1 was evaluated by peptide chip analysis. Since the role of Gal d 2 is controversially discussed,^7^ we decided to additionally map the linear epitopes of Gal d 3, another major egg white allergen of which little information is known so far.

## 2 METHODS

### 2.1 Patient selection

Patients (n = 35) sensitized to at least one of the recombinant hen's egg components rGal d 1 and rGal d 3, examined in 121 patients with hen's egg‐related sensitization (SPT, ImmunoCAP, ISAC) by Western Blot, were retrospectively selected from patients who visited the Dermatology/Allergology outpatient clinic of the University Medical Center (UMC) Utrecht between 2008 and 2018. These patients were divided into (a) hen's egg allergic (n = 16) and (b) hen's egg tolerant (n = 20) patients based on either double‐blind placebo‐controlled food challenge (DBPCFC) with heated egg or convincing history confirmed by a trained physician (challenged: 44% allergic group, 29% tolerant group). Convincing history was defined as immediate symptoms including oral allergy syndrome, skin reactions, gastrointestinal, respiratory or cardiovascular symptoms and an onset within 2 hours after ingestion. Gastrointestinal symptoms had to be combined with at least one additional immediate type symptom.

For the epitope discovery, sera from 13 allergic (8 suffering from objective symptoms) and 15 tolerant patients were applied on the peptide chip. Ethical approval (number 18‐428) was acquired from the biobank committee of the UMC Utrecht.

### 2.2 Heterologous expression of hen's egg components

The mature hen's egg components Gal d 1 (accession number: P01005) and Gal d 3 (accession number: P02789) were heterologously expressed as fusion proteins with N‐terminal‐His (6x)‐tag in *E coli* and purified as previously described.^12^, ^13^ All heterologously expressed proteins were purified by immobilized metal ion chromatography under denaturing conditions. Purified Gal d 1 and 3 were separated by gel electrophoresis and blotted onto a nitrocellulose membrane.

### 2.3 Determination of sIgE and sIgG4 sensitization

Sensitization to egg white and yolk extract was determined using the commercially available ImmunoCAP system and sIgE and sIgG4 sensitization to the native components Gal d 1, 2, 3 and 4 were measured using the EUROLINE‐immunoblot strip “Paediatrics’ 1” (DP 3812‐1601‐1 E, EUROIMMUN AG, Germany) according to manufacturer's instructions. Briefly, the immunoblots were manually incubated overnight at room temperature with serum diluted 1:11 (IgE) or 1:51 (IgG4) in working strength universal buffer (WSUB). After extensive washing with WSUB, bound IgE and IgG4 antibodies were detected with anti‐human IgE or IgG4 conjugate coupled with alkaline phosphatase. Upon another extensive washing step, visualization was provided by applying nitro‐blue tetrazolium/5‐bromo‐4‐chloro‐3’‐indolyphosphate substrate for ten minutes and specific IgE levels were evaluated as EUROLINE (EL)‐intensities and expressed as response units. Specific IgE levels to the heterologously expressed hen's egg components were determined under the same conditions.

### 2.4 Microarray design

A microarray with synthetic 15mer peptides, comprising the sequence of the mature Gal d 1 (accession number: P01005) and Gal d 3 (accession number: P02789) (offset = 3 due to limited space), was commercially obtained (PEPperPRINT). The peptide length of 15 amino acids was in accordance with the experience of PEPperPRINT to provide sufficient sensitivity without significant formation of secondary structures. All peptides were printed in triplicates with a linker consisting of 2 ß‐alanine and one aspartic acid. This linker was chosen to circumvent the binding of negatively charged fluorescent dyes to positively charged amino acids which are close to the array surface.

### 2.5 Microarray incubation

The microarray incubation was performed as previously described.^14^ Briefly, patient sera were diluted 1:4 in WSUB and incubated overnight. For detecting bound‐specific IgE and IgG4, a biotinylated anti‐IgE antibody (clone MHE‐18 1:5000, BioLegend) and simultaneously a biotinylated anti‐human IgG4 coupled with Neutravidin DyLight 680 (clone HP6025, 1:5000, Southern Biotech) were applied on the microarray and incubated for one hour at room temperature. Bound biotinylated human anti‐IgE antibodies were visualized by adding Neutravidin DyLight 800 (1:5000, Thermo Fisher) for one hour at room temperature. After extensive washing and drying, the microarray slides were scanned at a wavelength of 700 nm for IgG4 and 800 nm for IgE (intensity: 8.5) and the focus was set to 0.8 mm and the resolution to 21 µm.

### 2.6 Microarray evaluation

For data evaluation, the fluorescent signals for each peptide were obtained using the Pepslide Analyzer Software (SICASYS) with the fixed‐spot adjustment and the logarithmic signal‐to‐noise ratios (S) were computed according to the following quotation:

$$Si=log2\frac{TotalFluorescence(Peptide)}{BackgroundFluoresence(Peptide)}$$

For normalization, the S‐values were compared to the S‐values of blank spots, resulting in z‐scores defined as:

$$Zi=\frac{Si‐Median(SBlank)}{MedianDeviation(SBlank)}$$

Epitopes were defined as recognition of 2‐4 contiguous peptides with a median z‐score ≥ 3.0 and the amino acid residues are counted based on the amino acid sequence without signal peptide.

### 2.7 Determination of surfaced exposed epitopes

Surface‐exposed residues of an epitope were determined by submitting the 3D structure (Gal d 3, PDB ID: 1OVT) to the http://curie.utmb.edu/getarea.html interface.^15^ Under the conditions (default settings) as radius of the water probe set to 1.4 and no gradient in calculations, the algorithm calculates the probability of each residue to be solvent accessible. For an epitope, at least 25% of its residues must have a greater probability than 50% to be solvent accessible for calling this epitope “surface‐exposed.” The definition was confirmed by mapping the linear epitopes onto the 3D structure of Gal d 3 (pdb: 1OVT) using PyMol 1.3 (Schrödinger, Inc, USA). The corresponding images are shown in Figure S1.

### 2.8 Statistical analyses

Statistical differences between the hen's egg allergic and tolerant adults regarding their sensitization profiles were evaluated with the non‐parametric Mann‐Whitney U test and visualized by GraphPad Prism 8.3. For peptides and epitopes derived from Gal d 1, their recognition by IgE was evaluated by principle component analyses in R. Heat maps were generated in R using the “ComplexHeatmap” package.^16^

## 3 RESULTS

### 3.1 Patient characteristics

Patients sensitized to the recombinant components rGal d 1 and/ or rGal d 3 were divided into (a) allergic (75% female) and (b) tolerant patients (52% female) based on food challenge outcome or convincing history. Patients with subjective symptoms were more often diagnosed by a food challenge (4/6:67%) compared to patients with objective symptoms (3/7:43%), reducing at least the risk of misclassification. Allergic patients showed a median age of 25 and were overall younger than the tolerant patients with a median age of 28, although the age range was comparable (*P* = .63). Even though the majority of patients were co‐sensitized to nGal d 1 and nGal d 3, one allergic and one tolerant patient were mono‐sensitized to nGal d 1 and one tolerant patient was mono‐sensitized to nGal d 3. Interestingly, up to 94% of all included patients, irrespective of allergy or tolerance, suffered from atopic dermatitis. Besides, more than 60% of all included patients experienced symptoms related to allergic asthma (allergic 63%, tolerant 75%) and allergic rhinitis (allergic 81%, tolerant 60%). All characteristics are shown in Table 1 and File S1.

**Table 1:** Patient characteristics

	Allergic (n = 16)	Tolerant (n = 19)
Age (median [range])	25 [19‐65]	28 [19‐70]
Sex female [n, %]	12 [75%]	11 [58%]
Food challenge^a^ [n, %]	7 [44%]	5 [26%]
Symptoms [n, %]		
Objective	10 [63%]	NA
Subjective	6 [37%]	NA
No symptoms	NA	19
Sensitization [median, range]		
ImmunoCAP egg white	7.0 kU/L [0.6‐77 kU/L]	4.6 kU/L [0.5‐55 kU/L]
ImmunoCAP egg yolk	0.9 kU/L [0‐36 kU/L]	3.3 kU/L [0‐19.4 kU/L]
EUROLINE Gal d 1^b^	67 RU [20‐110 RU]	33 RU [0‐105 RU]
EUROLINE Gal d 2^b^	81 RU [28‐113 RU]	55 RU [6‐115 RU]
EUROLINE Gal d 3^b^	44 RU [0‐85 RU]	43 RU [0‐117 RU]
EUROLINE Gal d 4^b^	0 RU [0‐30 RU]	1 RU [0‐28 RU]
Co‐morbidities [n, %]		
Atopic dermatitis	15 [94%]	18 [95%]
Allergic asthma	10 [63%]	15 [79%]
Allergic rhinitis	13 [81%]	12 [63%]

Note

EUROLINE intensities expressed as response units (RU): <3 ≙ EAST‐class 0; 3‐6 ≙ EAST‐class 1; 7‐15 ≙ EAST‐class 2; 16‐30 ≙ EAST‐class 3; 31‐50 ≙ EAST‐class 4; 51‐100 ≙ EAST‐class 5; >100 ≙ EAST‐class 6.

^a^ Food challenges with heated hen's egg.

### 3.2 sIgE levels towards hen's egg extracts overlapped largely between allergic and tolerant but sensitized patients

**Figure 1:**
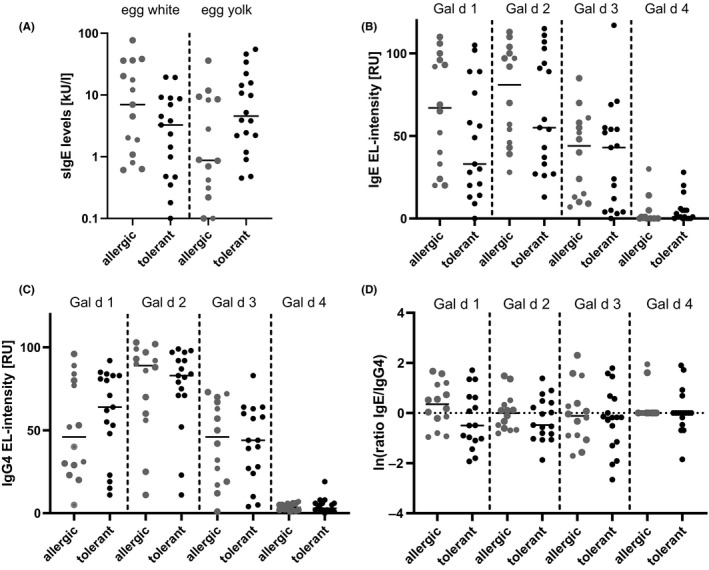
Sensitization profiles of tolerant and allergic patients to hen´s egg extracts and components. A, sIgE levels to egg white and egg yolk extract measured with the ImmunoCAP system (kU/L) split by allergic and tolerant patients; represented with the median. B, sIgE and sIgG4 levels to native hen's egg components Gal d 1, Gal d 2, Gal d 3 and Gal d 4 measured with the EUROLINE‐immunoblot (EL‐intensities, RU) split by allergic and tolerant patients; represented with the median. C, Log‐transformed sIgE/IgG4 ratios to native hen's egg components Gal d 1, Gal d 2, Gal d 3 and Gal d 4 resulting from the measures shown in b); represented with the median

All patients of this study were included based on their sensitization to at least one heterologously expressed hen's egg component (rGal d 1 and/or rGal d 3) and hence, detectable sensitization to hen's egg extracts was detected in all tested patients. Specific IgE titres towards egg white and yolk extract overlapped greatly between allergic and tolerant adults, resulting in low specificity even at increased cut‐off levels (0.53 at 5 kU/L). Overall, tolerant patients tend to have lower sIgE titres to egg white extract than allergic patients (median 4.6 kU/L vs 7.0 kU/L). On the other hand, sIgE levels to yolk extract were even higher in tolerant patients (median 3.3 kU/L) compared to allergic patients (median 0.9 kU/L), suggesting greater relevance of hen's egg white proteins compared to yolk‐derived ones (Figure 1A). No statistically significant difference was observed for egg white and egg yolk extract.

### 3.3 Gal d 1 sIgE/sIgG4 ratios were higher in allergic patients

As specific IgE levels towards hen's egg white extract were on average higher in allergic than in tolerant patients, we next analysed the relevance of antibodies against native components present in egg white. Although allergic and tolerant patients showed similar sensitization patterns towards nGal d 1, 2, 3 and 4 (Figure S2), the tolerant group showed a decreased median of 33 EL‐intensities towards nGal d 1 whilst allergic patients showed a median of 67 EL‐intensities (Figure 1B). On the other hand, the tolerant group showed an increased median of sIgG4 levels against nGal d 1 (tolerant: 71 EL‐intensities, allergic: 46 EL‐intensities), resulting in a decreased median of sIgE/sIgG4 ratios for nGal d 1 (median:0.5 vs 0.35, Figure 1B + C) in tolerant compared to allergic adults. The range, however, was comparable between both groups. Regarding nGal d 2, 3 and 4, also no statistically significant differences were observed.

### 3.4 Binding of sIgE to Gal d 3 was greatly reduced upon linearization

**Figure 2:**
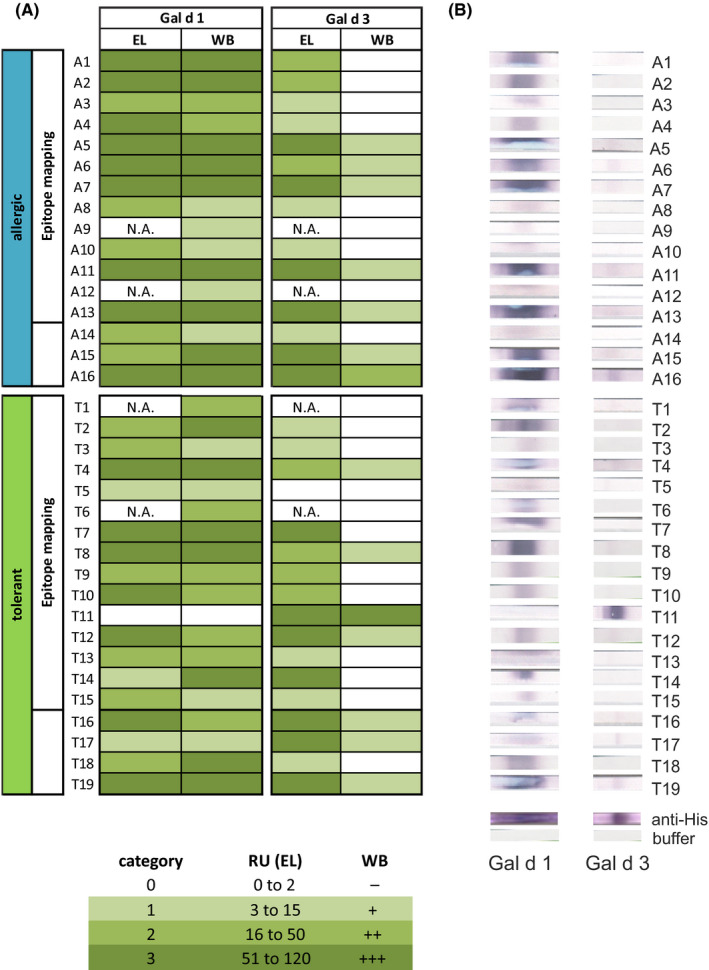
Linearization effects sIgE‐binding to Gal d 3. A, Comparison of IgE‐binding capacity between native (EL) and linearized Gal d 1 and Gal d 3 (WB); the number of bound IgE is divided into categories defined as low 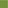, moderate 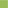 and high 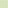. B, original WB showing sIgE‐binding to the recombinant components rGal d 1 and rGal d 3

Although nGal d 3 was recognized by lower sIgE titres compared to nGal d 1 and nGal d 2, nGal d 3 was recognized by all allergic patients and 93% of tolerant patients. As shown in Figure 2, the binding capacity of nGal d 3 was greatly reduced upon linearization (Western blot (WB)) of the heterologously expressed rGal d 3 compared to the native component. For instance, patient A6 reacted strongly to the native form of nGal d 3 while no binding to the linearized form was observed, indicating the importance of conformational epitopes for the recognition of Gal d 3 by IgE. In contrast, the binding capacity of rGal d 1 was hardly affected by linearization, pinpointing to the relevance of linear epitopes in recognizing Gal d 1.

### 3.5 Linear epitope recognition of Gal d 1 confirms clinically relevant sensitization in allergic patients with objective symptoms

**Figure 3:**
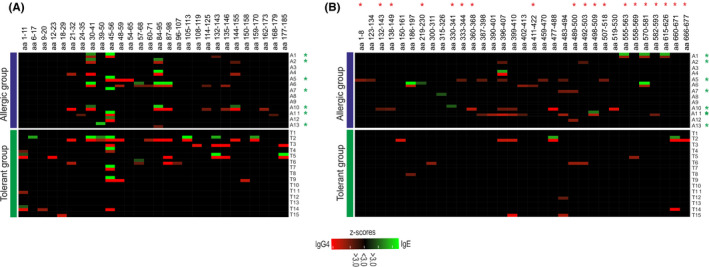
Peptide and epitope analysis derived from Gal d 1 and Gal d 3. A + B, Heatmap of Gal d 1 and Gal d 3 showing the IgE (green) and IgG4 (red) epitope recognition pattern of 13 hen's egg allergic and 15 hen's egg tolerant adults expressed as mean z‐scores. Epitopes recognized by sIgE and sIgG4 from the same individual are shown in rows underneath each other. Surface‐exposed epitopes of Gal d 3 are indicated with a red star and patients suffering from objective symptoms are highlighted with a green star

As linearization of rGal d 1 only marginally affected IgE‐binding, we next analysed linear epitope recognition with a peptide microarray. By means of principle component analysis with normalized values (z‐scores) for each peptide, one allergic patient was separated from the remaining patients within the first dimension and four allergic patients were separated within the second dimension. This effect was even more dominant by repeating the principle component analysis with the mean z‐scores of consensus sequences (epitopes) (Figure S3). Principle component 1 (dimension 1) was dominantly driven by epitope aa 45‐56 (97%) while principle component 2 was mainly driven by aa 30‐41 (49%) and aa 84‐95 (47%), having the greatest impact on discrimination. Accordingly to the principle component analysis, 6/13 allergic (46%)—4 suffered from objective symptoms—and 1/15 (7%) tolerant patient recognized at least one of the epitopes aa 30‐41, aa 39‐50 and aa 84‐95 as shown in Figure 3, indicating the potential of these epitopes to confirm clinically relevant sensitization to Gal d 1. Even though these findings have to be validated in a larger cohort, the recognition of these epitopes was highly specific (0.93) compared to sIgE titres to egg white extract (0.53 at 5 kU/L) or sIgE/sIgG4 ratios for Gal d 1 (0.63 at a ratio of 1).

Allergic patients who recognized aa 30‐41 combined with aa 84‐95 experienced respiratory symptoms (n = 2) or severe OAS (n = 1). The epitope aa 45‐56, known to be exclusively recognized by children with persistent hen's egg allergy,^8^ was recognized by 5 allergic (38%) but also by 4 tolerant patients (27%). Moreover, most of the epitopes recognized by IgE were simultaneously recognized by IgG4 derived from the same individual. However, 3 allergic patients (23%), all suffering from mild subjective symptoms as manifested by food challenges with heated hen's egg, did not recognize any epitope by either IgE or IgG4, suggesting a higher relevance of conformational epitopes in recognizing Gal d 1 by patients with mild symptoms.

### 3.6 Most patients with objective symptoms also recognized linear epitopes of Gal d 3

Additionally, a linear epitope mapping was also performed for Gal d 3. Regarding Gal d 3, only a small number of different epitopes (n = 11) were recognized by IgE in relation to its molecular mass (78 kDa) as already indicated by the reduction in sIgE‐binding upon linearization. In total, only nine different epitopes were recognized by six allergic patients while two epitopes were recognized by one tolerant patient (Figure 3). These epitopes bound by sIgE were mostly located on the surface of Gal d 3 and therefore easily accessible for antibody binding. Surface‐exposed epitopes were defined as possessing ≥ 3 residues (≥ 25%) facing the outside of the three‐dimensional structure (indicated with red stars in Figure 3). Three of the allergic patients and one tolerant recognized at least one surface‐exposed residue by sIgE. These patients, suffering from objective symptoms, were the same patients who recognized at least one of the linear epitopes which confirmed clinically relevant sensitization to Gal d 1. In contrast, IgG4 antibodies bound to a larger number (n = 30) of epitopes although most of them were just recognized by IgG4 from one to two individual patients except for the epitopes aa 396‐407 and 483‐494 not located on the protein surface.

## 4 DISCUSSION

So far, sensitization to hen's egg extracts, components and linear epitopes is solely studied in children although persistent and newly onset hen's egg allergy do appear in adults with a prevalence of 0.02 to 0.6% across European countries.^1^, ^2^ In the present study, we showed great overlap in sIgE levels to hen's egg extracts or single components between allergic and tolerant, but sensitized adults. Clinically relevant sensitization to Gal d 1 was confirmed by sIgE‐binding to the linear epitopes aa 30‐41, aa 39‐50 or aa 84‐95 in 6 out of 13 hen's egg allergic adults, mainly suffering from objective symptoms. In contrast, patients with mild subjective symptoms showed no binding to linear epitopes of Gal d 1.

This is, to our knowledge, the first study focussing on sensitization patterns in hen's egg allergic and tolerant adults. While largely overlapping sIgE titres to egg white extract were not able to clearly discriminate between allergy and tolerance in adults, the definition of clinically relevant cut‐off levels appeared to be supportive in diagnosing raw or heated hen's egg allergy in children.^5^, ^17^, ^18^, ^19^ Despite lacking the complete information about heated egg tolerance in our study population, a similar tendency of higher sIgE titres to egg white extract was observed in allergic (median: 7.0 kU/L) compared to tolerant adults (median: 4.6 kU/L). Although our cohort selection based on sensitization towards at least one heterologously expressed hen's egg component (rGal d 1 and/or rGal d 3) resulted in a certain selection bias, overlapping sIgE titres to Gal d 1 (up to 10 kU/L) and therefore to egg white extract, containing Gal d 1 as major allergen, were also described in hen's egg allergic and tolerant children.^20^

While IgE‐binding to Gal d 3 strongly decreased upon linearization (SDS‐PAGE under reducing conditions and western blotting), the IgE‐binding to Gal d 1 was only slightly altered, pinpointing to the importance of linear epitopes for the recognition of Gal d 1. Nevertheless, 23% of the allergic (subjective symptoms) and 53% of the tolerant patients who showed sIgE‐binding to the linearized form of Gal d 1 did not show any sIgE‐binding towards linear epitopes on the microarray, suggesting incomplete linearization of Gal d 1 potentially due to reduced accessibility of disulphide bridges by reducing agents.^21^, ^22^ Incomplete linearization and the lack of linear epitope recognition in a part of the patients pinpoint to the importance of conformational epitopes for the recognition of Gal d 1 in these patients. A similar observation was made by Martínez‐Botas and colleagues where 34% of hen's egg allergic children strongly positive to Gal d 1 did not recognize any linear epitope by sIgE,^10^ suggesting exclusive recognition of conformational epitopes in a subpopulation of hen's egg allergic patients.

The epitopes aa 30‐41, aa 39‐50 and aa 84‐95 were mostly recognized by allergic patients (6/13 allergic vs 1/15 tolerant patients) who suffered from objective symptoms (4/7) upon hen's egg ingestion, confirming clinically relevant sensitization to Gal d 1 despite overlapping sIgE titres between allergic and tolerant adults. Although these epitopes were described independently in different studies with hen's egg allergic children,^9^, ^11^, ^23^ they did not belong to the so‐called “informative” epitopes (aa 1‐10, aa 11‐20, aa 47‐56 and aa 113‐122) which showed great potential to predict persistent hen's egg allergy in children.^8^ The epitope aa 47‐56, however, was recognized by 38% of allergic vs 27% of tolerant adults (overall 32% of all patients) in the present study, suggesting divergent IgE specificities in adulthood compared to childhood. These differences may also be related to study design and inclusion criteria.

Notably, epitopes recognized bys IgE were often simultaneously bound by sIgG4 from the same individual irrespectively of their status, suggesting a clonal relationship between the sIgE and sIgG4 antibodies. A clonal relationship would imply that IgE‐producing B cells partially originate from IgG4 + B cells or that some IgE + and IgG4 + B cells share the same origin. Clonal analysis of allergic patients showed a predominately clonal relationship between IgG1 + and IgE + B cells, suggesting that IgG1 + B cells might be the shared origin.^24^ Compared to children, this potential clonal relationship between sIgE and sIgG4 appeared to be less dominant, since sIgE‐binding overlapped only partially with the binding characteristics of sIgG4.^10^ This discrepancy between children and adults regarding potential clonal relationship between IgE + and IgG4 + B cells might indicate the alteration of the origin for IgE + and IgG4 + B cells over time. However, more research is needed to confirm this hypothesis.

As a step forward, the promising results of the present study should be validated in a prospective cohort exclusively diagnosed by food challenge, minimizing the risk of misclassification. Moreover, inclusion based on suspicion of egg allergy provides a broader population with more distinct sensitization patterns such as mono‐sensitization to Gal d 2.

In conclusion, sIgE‐binding to linear epitope of Gal d 1 (aa 30‐41, aa 39‐50 or aa 84‐95) is highly specific to identify hen's egg allergic adults, mainly suffering from objective symptoms and may improve sIgE diagnostics as an additional tool to conventional testing using egg extracts and single allergen components.

## CONFLICT OF INTEREST

E. Wierzba, U. Flügge and W. Suer are employees of EUROIMMUN AG, Lübeck, Germany. The research position of A. Ehlers is partially funded by EUROIMMUN AG, Lübeck, Germany. Other authors have no conflicts of interest to declare.

## AUTHOR CONTRIBUTIONS

AE, HO, EW, UF and WS involved in experimental design; AE, AK and TML involved in patient selection; AE and EW involved in experimental performance; AE, EW, UF and WS involved in data collection and analyses; AE, HO, EW, UF, AC and WS involved in contribution to data interpretation; AE involved in drafting the manuscript; HO, EW, UF, TML, AK and WS: involved in critical revision of the manuscript.

## INFORMED CONSENT

This study was carried out in accordance with the University Medical Center Utrecht, Biobank Regulations, which are in compliance with the applicable national and international laws and regulations. These regulations permit the use of “residual material from diagnostic testing” for research, unless the patient objects (Article 8, “no objection” procedure). None of the included patients objected the use of their serum. The protocol was approved by the Biobank Research Ethics Committee of the University Medical Center Utrecht under the protocol number 18‐428.

5

## Supporting information

Supplementary Material

Supplementary Material

Supplementary Material

## Data Availability

All data generated or analysed during this study are included in this published article and its supplementary information files.
