# Supplementary material for: Detection of specific IgE against linear epitopes from Gal d 1 has additional value in diagnosing hen’s egg allergy in adults
Source: Clin Exp Allergy. 2020 Sep 24;50(12):1415–23. doi: 10.1111/cea.13730 (PMC7756380; doi:10.1111/cea.13730)
Supplement: Supplementary file 3 — Supplementary Material [file CEA-50-1415-s003.docx]

**Supplementary File 3**

**Table S1:** Comparison of recognised epitopes of Gal d 1 with previous described epitopes

| Peptide | | Residues  (without signal sequence) | IgE | IgG4 | Publication  IgE* |
| --- | --- | --- | --- | --- | --- |
| AEVDCSRFPNA | 1-11 | | X | X | 1,2,4 |
| SRFPNATDKEGK | 6-17 | | X |  | 1,5 |
| PNATDKEGKDVL | 9-20 | |  | X | 1,4,5 |
| TDKEGKDVLVCN | 12-23 | |  | X | 2 |
| DVLVCNKDLRPI | 18-29 | |  | X |  |
| VCNKDLRPICGT | 21-32 | |  | X |  |
| KDLRPICGTDGV | 24-35 | |  | X |  |
| CGTDGVTYTNDC | 30-41 | | X | X | 2,3,4 |
| NDCLLCAYSIEF | 39-50 | | X | X | 3,4 |
| AYSIEFGTNISK | 45-56 | | X | X | 1,4,5 |
| IEFGTNISKEHD | 48-59 | |  | X |  |
| ISKEHDGECKET | 54-65 | |  | X | 2,3 |
| EHDGECKETVPM | 57-68 | | X | X | 2,4 |
| GECKETVPMNCS | 60-71 | |  | X |  |
| *CSSYAN* | *71-75* | |  |  | *3* |
| *DGKVMVLCNRA* | *80-90* | |  |  | *3* |
| MVLCNRAFNPVC | 84-95 | | X | X | 1,4 |
| CNRAFNPVCGTD | 87-98 | |  | X |  |
| GTDGVTYDNECL | 96-107 | |  | X | 3,5 |
| ECLLCAHKV | 105-113 | | X | X | 2,4 |
| LCAHKVEQGASV | 108-119 | |  | X |  |
| EQGASVDKRHDG | 114-125 | |  | X | 4 |
| *KRHDGGCRKELAAV* | *121-134* | |  |  | *2,3* |
| AAVSVDCSEYPK | 132-143 | | X | X | 4 |
| SVDCSEYPKPDC | 135-146 | |  | X |  |
| PDCTAEDRPLCG | 144-155 | | X | X |  |
| DRPLCGSDN | 150-158 | |  | X | 4 |
| KTYGNKCNFCNA | 159-170 | | X | X | 3 |
| GNKCNFCNAVVE | 162-173 | |  | X | 3 |
| CNAVVESNGTLT | 168-179 | |  | X |  |
| TLTLSHFGK | 177-185 | | X | X | 1,3 |

*****1: Cooke et al. 1997; 2: Holen et al. 2001; 3: Mine and Zhang 2002; 4: Järvinen et al. 2007, red=informative; 5: Martínez-Botas 2013

**
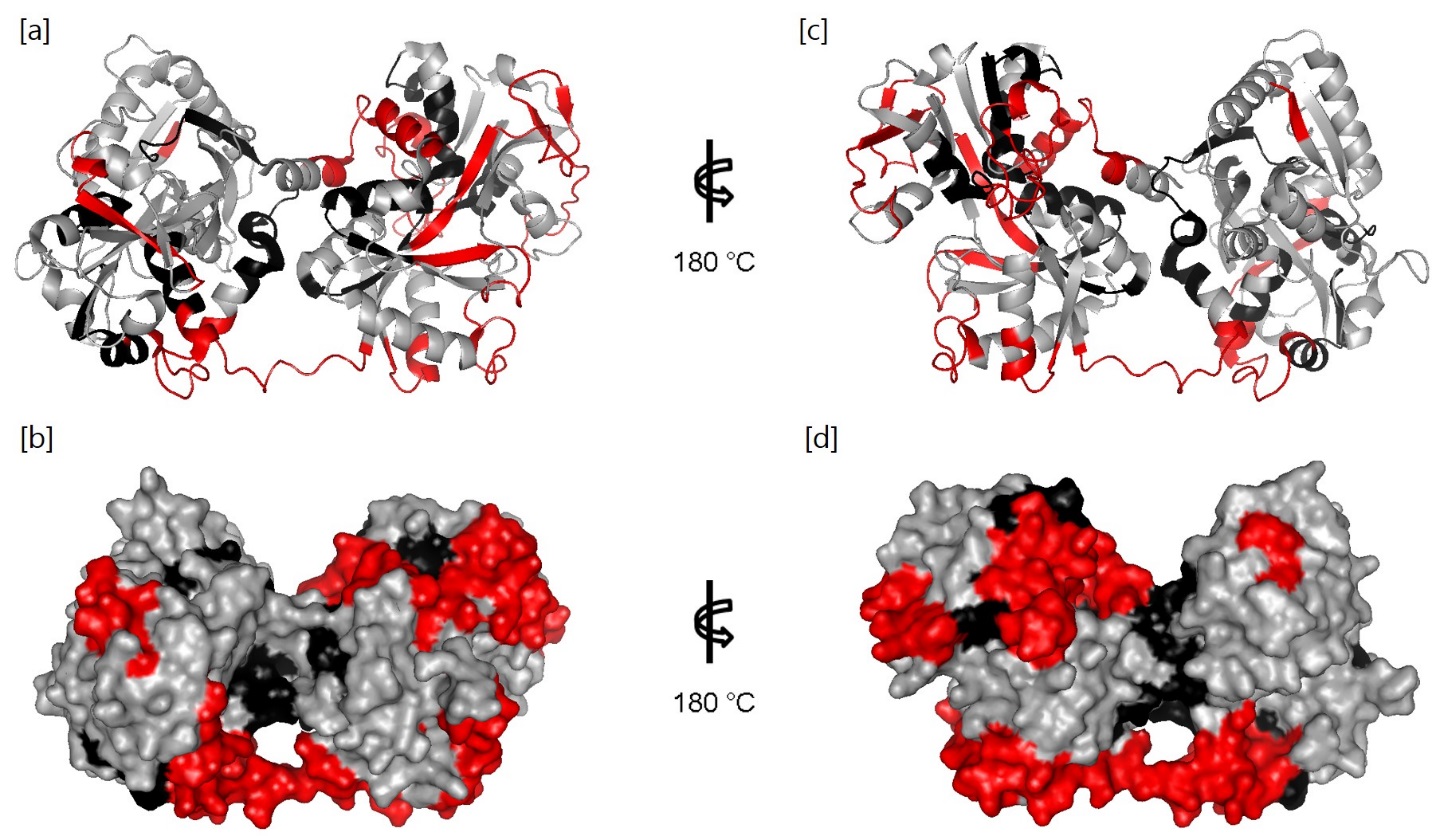
**

**Figure S1:**

The as surface-exposed (red) and non-surface exposed (black) defined epitopes were mapped onto the 3D structure of Gal d 3 (pdb: 1OVT).

[a] Cartoon view highlighting the dominant location of as surface-exposed defined epitopes in the loops of  Gal d 3.

[b] View on the solvent-accessible surface of Gal d 3

[c] Cartoon view as in [a] but turned for 180 °

[d] View on the solvent-accessible surface of Gal d 3 as in [b] but turned for 180 °

**Figure S2:** Sensitisation profiles of hen’s egg allergic and tolerant patients to the native hen’s egg components Gal d 1, Gal d 2, Gal d 3 and Gal d 4 are separately shown

**
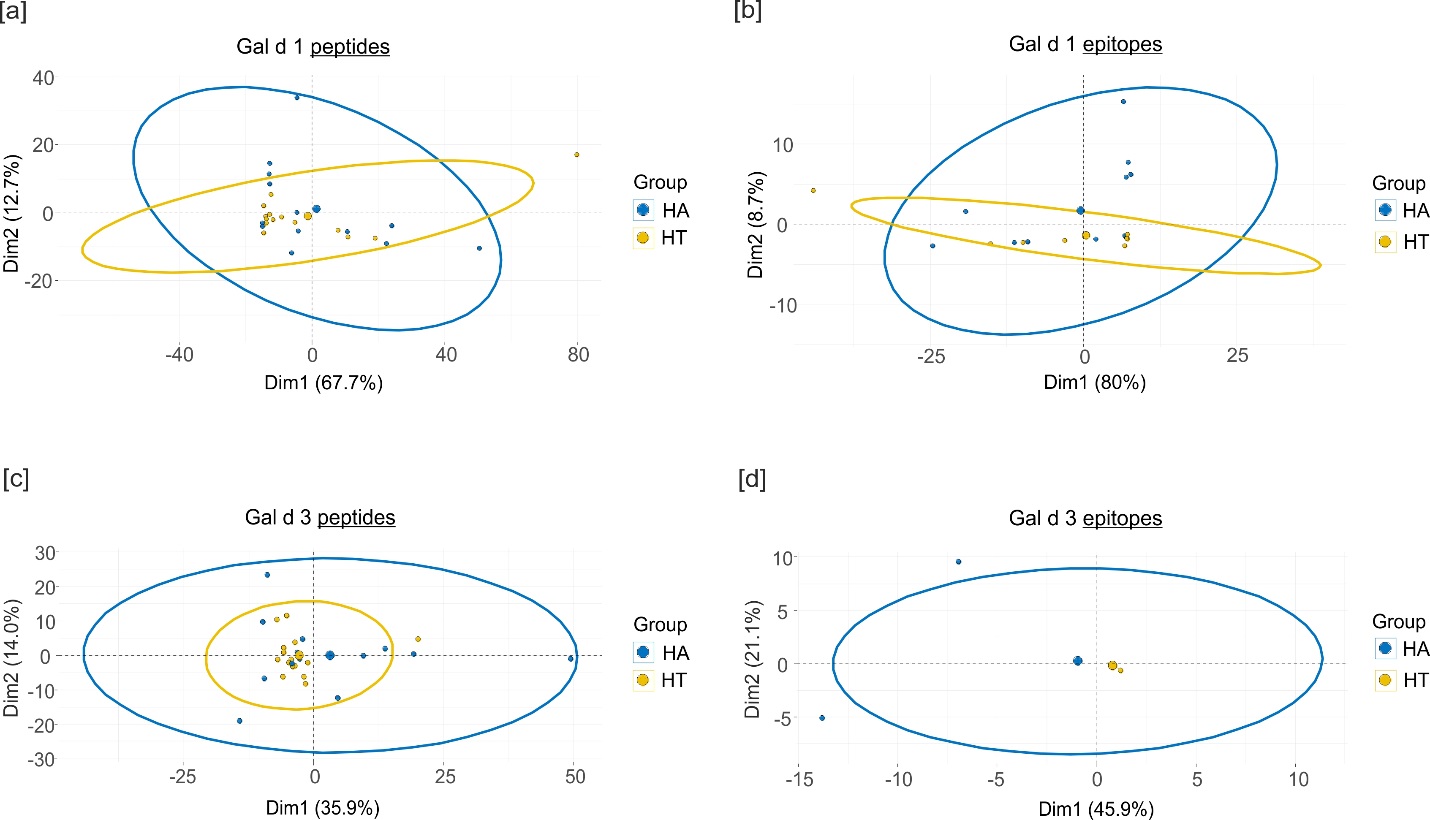
**

**Figure S3:**

(a) Principle component analysis of peptides derived from Gal d 1 recognised by IgE and (b) principle component analysis of epitopes derived from Gal d 1 recognised by IgE

(c) Principle component analysis of peptides derived from Gal d 3 recognised by IgE and (d) principle component analysis of epitopes derived from Gal d 3 recognised by IgE
